# An ICF-Based Model for Implementing and Standardizing Multidisciplinary Obesity Rehabilitation Programs within the Healthcare System

**DOI:** 10.3390/ijerph120606084

**Published:** 2015-05-29

**Authors:** Amelia Brunani, Alberto Raggi, Anna Sirtori, Maria Elisa Berselli, Valentina Villa, Francesca Ceriani, Stefania Corti, Matilde Leonardi, Paolo Capodaglio, ICF-OBESITY Group

**Affiliations:** 1 Department of Rehabilitation Medicine, San Giuseppe Hospital, IRCCS Istituto Auxologico Italiano, Piancavallo, 28921 Verbania, Italy; 2 Neurology, Public Health and Disability Unit—Scientific Directorate, Neurological Institute C. Besta IRCCS Foundation, Milan, Italy; 3 Psychology Research Laboratory, San Giuseppe Hospital, IRCCS Istituto Auxologico Italiano, 28921 Verbania, Italy

**Keywords:** obesity, Rehab-CYCLE, ICF, rehabilitation

## Abstract

*Introduction/Objective*: In this study, we aimed to design an ICF-based individual rehabilitation project for obese patients with comorbidities (IRPOb) integrated into the Rehab-CYCLE to standardize rehabilitative programs. This might facilitate the different health professionals involved in the continuum of care of obese patients to standardize rehabilitation interventions. *Methods*: After training on the ICF and based on the relevant studies, ICF categories were identified in a formal consensus process by our multidisciplinary team. Thereafter, we defined an individual rehabilitation project based on a structured multi-disciplinary approach to obesity. *Results*: the proposed IRPOb model identified the specific intervention areas (nutritional, physiotherapy, psychology, nursing), the short-term goals, the intervention modalities, the professionals involved and the assessment of the outcomes. Information was shared with the patient who signed informed consent. *Conclusions*: The model proposed provides the following advantages: (1) standardizes rehabilitative procedures; (2) facilitates the flow of congruent and updated information from the hospital to outpatient facilities, relatives, and care givers; (3) addresses organizational issues; (4) might serve as a benchmark for professionals who have limited specific expertise in rehabilitation of comorbid obese patients.

## 1. Introduction

Obesity has been shown to play an important role in the trends in disability over the last two decades and in the dramatic increase in related chronic conditions [1]. It can account for increased limitations during activities of daily living (ADLs) and instrumental activities of daily living (IADLs), such as showering, dressing, indoor walking, as well as family activities, driving, working, manual lifting, and sports. The rates of musculoskeletal conditions, such as arthritis, back, neck, hip and knee problems, and comorbidities including diabetes, hypertension and heart disease (coronary heart disease and ischemic stroke), are higher in obese patients and frequently cited as causes of limitation in the ability to participate in social and occupational activities [2]. The presence of physical functional limitation due to obesity is not related to obesity degree (measured as BMI), gender or age [3].

Achieving sufficient weight loss to improve some clinical conditions is only part of a comprehensive rehabilitation program for obese patients with comorbidities. Treating obese patients, particularly those with severe obesity, implies assessing and staging all of the current conditions, functional limitations (physical, psychological and cognitive), taking into consideration additional factors such as the patient’s family and social environment. In a rehabilitative perspective and in the wake of the latest definition of Rehabilitation by the World Health Organization (WHO) [4], obese patients require a holistic rather than just a treatment approach to their care. A consensus document for the Rehabilitation of obese patients with comorbidities has been recently published [5], in line with the recommendations of the Italian Ministry of Health on the rehabilitative pathways [6]. The latter state that the Individual Rehabilitative Project (IRP) is the requisite to access rehabilitation care in Italy and constitutes the reference for all health professionals, including social care workers. The IRP is tailored to the individual needs and contains the prognosis, the patient’s expectations and priorities and his/her relatives/care givers, also defining the appropriateness and congruity of the different interventions. It also describes whether the completion of the rehabilitation phase has been successful in terms of the goals achieved. The specific intervention areas, the short-term goals, the modalities of application of the interventions, the professionals involved and the assessment of the results are exhaustively defined. In line with the “biopsychosocial model”, the ICF [7] has the aim of capturing and appropriately describing the health status at the individual and population levels. Previous experiences with the use of ICF in obesity [8,9] have provided a validated checklist for describing the functional profile of obese patients. Patients with obesity show several limitations in their body functions (B7 neuro-musculoskeletal and movement-related functions, B2 pain, B4 function of the cardiovascular), activities and participation (D5 self-care, D2 general tasks and demand, D7 relationships) and reported scarce environmental facilitators (E3 support)[10].

The Rehab-CYCLE [11] is a problem solving approach to rehabilitation management that integrates the universal model of the ICF within the rehabilitation cycle, where all professionals involved in the care of the patient coordinate their actions. Indeed, in the clinical practice, an integration between the interventions provided at different times by the various health professionals involved is often difficult both during the rehabilitation phase and especially when patients return home, where relatives, care-givers and general practitioners need to have the updated picture of their functioning and needs. With this in mind, after having previously defined an IRP specific for obese patients with comorbidities [5], in the present study, we have proposed a standardized IRP model for obese patients with comorbidities (IRPOb) in line with the Rehab-CYCLE approach that can integrate the information obtained from the ICF checklist, as in Raggi *et al.* [10]. The model might serve as a standard for rehabilitation interventions in obesity and offer suggestions for maintaining positive lifestyle changes within the community.

## 2. Methods

At our Institute, we had previously developed a Diagnostic and Therapeutic Protocol (DTP) to define the rehabilitation pathway of obese patients with comorbidities [12]. Its development relied on the contributions of rehabilitation specialists, endocrinologists and other medical specialists, nurses, physiotherapists, dietitians, psychologists and social care workers. Each professional performed his/her own evaluation using specific instruments, questionnaires, tests or clinical interviews. A preliminary study reported the methodological procedure for mapping our structured multi-disciplinary approach [9] onto the ICF manual published by WHO that contains the explanation for each ICF code [7]. After following the international training procedures on how to use the ICF and relying on the standardized set of coding rules, we linked the mapping to a checklist of 166 ICF categories [9], expanding and modifying the previous core set for obesity containing 157 ICF categories published by Stucki *et al.* [8]. In a subsequent clinical study [10], we analyzed data obtained from 51 obese patients and validated the checklist with the aim of identifying the characteristics of the obesity-related disability.

**Figure 1:**
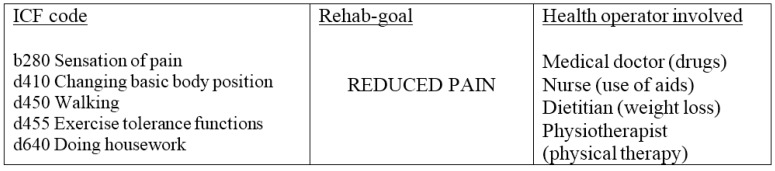
Example of the decision-making process in Individual Rehabilitation Project for obesity (IRPOb).

**Figure 2:**
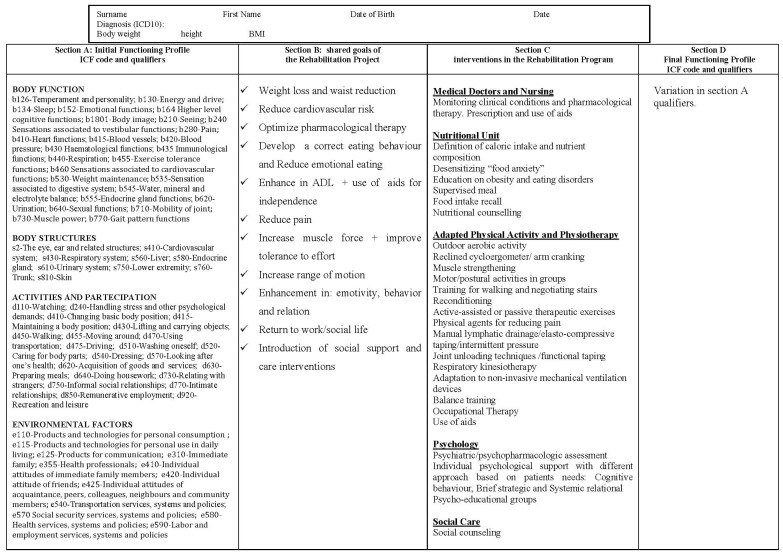
The Individual Rehabilitation Project for obesity (IRPOb) and its specific interventions (Program).

The evaluation of the disability profile obtained from our checklist, represents the starting point for the formulation of the IRP, which includes the definition of specific interventions (nutrition, physiotherapy, psychological support, nursing), short-term goals, intervention modalities, healthcare professionals involved and outcome assessments, all of which have to be shared with the patient. Figure 1 shows an example of the decision-making process in IRPOb, where the functional diagnoses obtained from the ICF checklist are implemented. All members of the ICF-trained and obesity expert team were involved in formal and consensus process. This stage involved integrating the clinical evidence into the treatment of obesity, the consensual definition of all possible outcomes (Figure 2, Section B) and the specific interventions to achieve the short-term goals. Two multidisciplinary groups within the team discussed the feasibility of applying the IRPOb to a Rehab-CYCLE [11]. To complete the Rehab-CYCLE, we also defined the outcomes based on benchmark results as described in Precilios *et al.* [13]. The Institute’s Ethics Committee approved the study and each patient signed a written consent form.

## 3. Results

Figure 2 shows the model of the ICF-based Individual Rehabilitation Project for obesity (IRPOb). Section A of this format describes the patient’s functional profile using the ICF codes and qualifiers. Based on our previous results using ICF for assessing obesity-related disability [9,10,14], we have depicted here the categories in which at least 20% of our patient population did report a problem relevant for their functional profile. Section B provides the definition of the long-term goals of the rehabilitation project shared with the client. Section C describes the specific interventions required to achieve these goals (Rehabilitation Program). Section D shows the functional status changes after the interventions. All sections have to be filled in by each healthcare professional, using the ICF qualifiers, according to their specific competences.

## 4. Discussion

The evaluation and treatment of patients with disabling obesity requires clinical facilities where they can be treated with appropriate therapeutic and rehabilitative protocols. It is important to develop rehabilitation pathways based on a multidisciplinary approach not only dealing with the weight issue in the long term but, also, preventing and treating complications. The Individual Rehabilitation Project (IRP) defined the by the Italian Ministry of Health represents in our view the starting point for defining a model to be implemented in the clinical practice [6]. This document recommends ICF classification for defining health status and functional profile. The practical implementation of ICF in rehabilitation, however, appears limited due to the need of a multidisciplinary approach in the diagnostic evaluation and in formulating the IRP. With this in mind, we aimed to propose the IRPOb model that, in line with the Rehab-CYCLE, integrates the IRP with an ICF-based individual evaluation of obese patients with comorbid conditions.

As previously reported [8,14], ICF evaluates obesity-related disability according to the degree of obesity and the associated conditions. The ICF can capture unhealthy habits and environmental factors that contribute to the maintenance of the obesity status. In line with previous experimental studies [15,16,17,18], the IRPOb offers the advantage of standardizing rehabilitative procedures in the realm of obesity with comorbidities and of facilitating the transfer of information from rehabilitation professionals within the hospital to out-patient facilities, families, caregivers, family doctors, social care workers and workplaces. Clinicians who do not frequently admit obese patients for rehabilitation may benefit from the information provided and patients will be granted quality of care from benchmark intervention procedures. Standardization also offers health operators an opportunity to address organizational issues. Bearing in mind that obesity, particularly severe obesity, is a chronic disease that has a significant impact on quality of life, clinical, psychological and social support are necessary to minimize disability. Sharing rehabilitation goals with the patients is a requisite for promoting self-care and long-term lifestyle changes.

The IRPOb monitors short-term results as well as events that may occur at follow-up for possible amendments to the continuum of care or lifestyle interventions. Evaluation of the long-term results includes: (i) goal achievement (e.g., weight loss, pain reduction, enhanced mobility); (ii) improvement in the patient’s overall clinical condition and (iii) reduction in disability progression.

From a public health perspective, the IRPOb might serve as a benchmark for hospitals admitting multiple morbid patients for rehabilitation but whose health personnel has limited experience with. Considering the complexity of comorbid obese patients, the IRPOb may represent a tool for tuning appropriate, consistent and effective rehabilitation interventions targeted on the real patients’ needs at that particular moment. Rehabilitation units with optimal standards of care, structurally and technologically adequate for the care of patients with morbid obesity, especially after an acute event, need to provide user friendly and useful information for the continuum of care to relatives, care givers, general practitioners or facilities providing rehabilitation at lesser intensity. In this light, IRPOb might well represent a facilitator to enhance quality and effectiveness of care in the long term. When changing the setting of care, the IRPOb may provide the professionals involved and the patient himself with correct and updated information regarding functional and health status and related needs for specific interventions.

The application of the ICF in rehabilitation maybe limited by the need for a time-consuming multidisciplinary approach for the diagnostic evaluation and design of the rehabilitation project. Larger studies will be required to evaluate the organizational impact of implementing the IRPOb in clinical practice and to confirm its clinical usefulness. Obesity is a chronic condition which may become highly disabling and costly for the National Health System. In this light, promoting possible ameliorations (methods, tools, organization and health costs reimbursement) of the clinical governance for rehabilitation programs represents a priority in this area.

## 5. Conclusions

We designed an ICF-based IRP for obese patients with comorbidities integrated into the Rehab-CYCLE to standardize rehabilitative programs for obese patients with comorbidities. In our experience, the multidisciplinary evaluation performed by an ICF-trained team contributes to highlight the key areas necessitating intervention. The IRPOb also emphasizes the consensual definition of goals and provides feedback on patient compliance with the project and the specific interventions. The lack of effective weight management programs, poor long-term patient compliance and the gaps in existing health services for the care of chronic conditions are all existing negative factors in daily practice. Filling those gaps by facilitating daily communication among health professionals and providing a flow of accurate information to be used at follow-up and in the continuum of rehabilitative care from hospital to families and care givers may represent one of the cornerstones of a more holistic approach to tackling disabling obesity.
